# Variegated tropical landscapes conserve diverse dung beetle communities

**DOI:** 10.7717/peerj.3125

**Published:** 2017-04-04

**Authors:** Cristiane Costa, Victor Hugo F. Oliveira, Rafaella Maciel, Wallace Beiroz, Vanesca Korasaki, Julio Louzada

**Affiliations:** 1 Setor de Ecologia/Departamento de Biologia, Universidade Federal de Lavras, Lavras, MG, Brazil; 2 Lancaster Environment Centre, Lancaster University, Lancaster, United Kingdom; 3 Departamento de Entomologia, Universidade Federal de Lavras, Lavras, MG, Brazil; 4 Departamento de Ciências Exatas e da Terra, Universidade do Estado de Minas Gerais, Frutal, MG, Brazil

**Keywords:** Biodiversity conservation, Agriculture, Countryside, Hedgerow, Landscape modification, Scarabaeinae, Forest fragments, Forest corridors

## Abstract

**Background:**

Conserving biodiversity in tropical landscapes is a major challenge to scientists and conservationists. Current rates of deforestation, fragmentation, and land use intensification are producing variegated landscapes with undetermined values for the conservation of biological communities and ecosystem functioning. Here, we investigate the importance of tropical variegated landscapes to biodiversity conservation, using dung beetle as focal taxa.

**Methods:**

The study was carried out in 12 variegated landscapes where dung beetles were sampled using six pitfall traps, 30 m apart from each other, along a transect in each studied landscape use and cover classes—LUCC (forest fragment and corridor, coffee plantation, and pasture). We baited each pitfall trap with 30 g of human feces and left open for a 48 h period. We also measured three environmental variables reflecting structural differences among the studied classes: canopy cover, local vegetation heterogeneity and soil sand content.

**Results:**

We collected 52 species and 2,695 individuals of dung beetles. We observed significant differences in the mean species richness, abundance and biomass among classes, with forest fragments presenting the highest values, forest corridors and coffee plantations presenting intermediate values, and pastures the lowest values. Regarding community structure, we also found significant differences among classes. Canopy cover was the only variable explaining variation in dung beetle species richness, abundance, biomass, and community structure. The relative importance of spatial turnover was greater than nestedness-resultant component in all studied landscapes.

**Discussion:**

This study evaluated the ecological patterns of dung beetle communities in variegated tropical landscapes highlighting the importance of these landscapes for conservation of tropical biodiversity. However, we encourage variegation for the management of landscapes that have already been fragmented or as a complementary initiative of current conservation practices (e.g., protection of natural habitats and establishment of reserves).

## Introduction

Conserving biodiversity in tropical landscapes is a major challenge to scientists and conservationists (Tilman et al., 2011). The tropics sustain most of the world’s described biodiversity (Brown, 2014), but suffer from the highest rates of deforestation and land use intensification—mainly due to rapid agricultural expansion (Wright & Muller-Landau, 2006; Gibbs et al., 2010). This scenario yields mosaics of multiple artificial/semi-natural areas of land use abruptly (fragmentation) or gradually (variegation) bordering natural habitats (Fischer & Lindenmayer, 2007), often resulting in the loss of native species (Arroyo-Rodriguez et al., 2013) and, in some cases, local extinctions (Newmark, 1991; Lehtinen & Ramanamanjato, 2006). Such a depletion of biodiversity can result in biotic homogenization (Solar et al., 2015) and, consequently, alter ecosystem functioning, leading to deterioration in the provisioning of ecosystem goods and services (Olden et al., 2004; Clavel, Julliard & Devictor, 2011; Mitchell et al., 2015).

Nevertheless, human modified landscapes are still useful for species conservation (Chazdon et al., 2009; Gardner et al., 2009), especially if patches of natural vegetation are present (Nichols et al., 2007; Fahrig, 2013), and if the matrix is highly suitable for local biodiversity (Prugh et al., 2008; Franklin & Lindenmayer, 2009). Consequences of human activities may differ between fragmented and variegated landscapes. While fragmented landscapes generally host isolated populations in habitat patches surrounded by hostile matrices (Fahrig, 2003; Fahrig et al., 2011), variegated landscapes present multiple artificial or semi-natural land uses that are gradually different from the natural habitats (McIntyre & Hobbs, 1999; Fischer & Lindenmayer, 2007). These landscapes may be more permeable to species movement, exhibiting distinct biodiversity patterns in response to human activities (Daily, 2001; Rös, Escobar & Halffter, 2012), and thus have high conservation value (Barlow et al., 2010; Gibson et al., 2011).

Nevertheless, studies on the responses of biodiversity to tropical variegated landscapes with large number of replicates are scarce (Fischer & Lindenmayer, 2007). The few related studies indicate that variegated landscapes are, in fact, more connected than fragmented landscapes and have variable importance for biodiversity conservation, sustaining high levels of biodiversity in the tropics (Barlow et al., 2007; Rös, Escobar & Halffter, 2012). However, these studies are generally conducted in recently modified areas that are under great influence from natural habitats (Barlow et al., 2010). Thus, it is difficult to disentangle the contribution of modified areas from that of natural habitats to biodiversity conservation. The investigation of older tropical variegated landscapes may offer important insights about future biodiversity patterns in these areas. Also, considering that most of the world’s terrestrial ecosystems and one quarter of world’s threatened species are living outside protected areas, understanding the importance of these human modified landscapes for biodiversity conservation becomes crucial (Rodrigues et al., 2004; Jenkins & Joppa, 2009; Troupin & Carmel, 2014; Ekroos et al., 2016).

Here, we investigated biological communities present in tropical variegated landscapes that have been subject to intense pressures of urbanization, agriculture and livestock production since the 18th century (ca. 300 years) (Zemella, 1990; Vilela, 2007). Our studied area is composed of mosaics of semi-deciduous secondary forest fragments (of variable sizes and regeneration status), native and introduced pasturelands, monocultures (mainly coffee plantations), and hedgerows (forest corridors) (Burel, 1996; Oliveira-Filho & Fluminhan-Filho, 1999; Castro & Van den Berg, 2013). We aimed to assess dung beetle communities in twelve 300-year-old tropical variegated landscapes in order to find empirical evidence of their conservation value to biodiversity. We used dung beetles as our focal taxa because species of this group are abundant in our studied area, easily sampled and identified, play important ecological roles, are associated with vertebrates, and are widely used as bioindicators (Nichols et al., 2007; Nichols et al., 2008; Nichols & Gardner, 2011; Gillett et al., 2016). Furthermore, dung beetle communities from tropical forests are greatly influenced by vegetation structure, due to their association with specific climatic (physiological intolerance) and edaphic conditions (Halffter & Arellano, 2002; see Nichols & Gardner, 2011 and references therein; Griffiths et al., 2015).

We investigated the extent to which dung beetle species richness, abundance and biomass, and community structure are affected by (1) land use and cover class—LUCC (i.e., forest corridors, forest fragments, coffee plantations and pastures), and (2) structural differences among habitats (i.e., variation in canopy cover, local vegetation heterogeneity and soil sand content). We also assessed the importance of landscape variegation to conservation of dung beetle regional diversity (3), disentangling the relative contribution of nestedness-resultant and spatial turnover to beta-diversity patterns in variegated landscapes.

## Material & Methods

**Figure 1:**
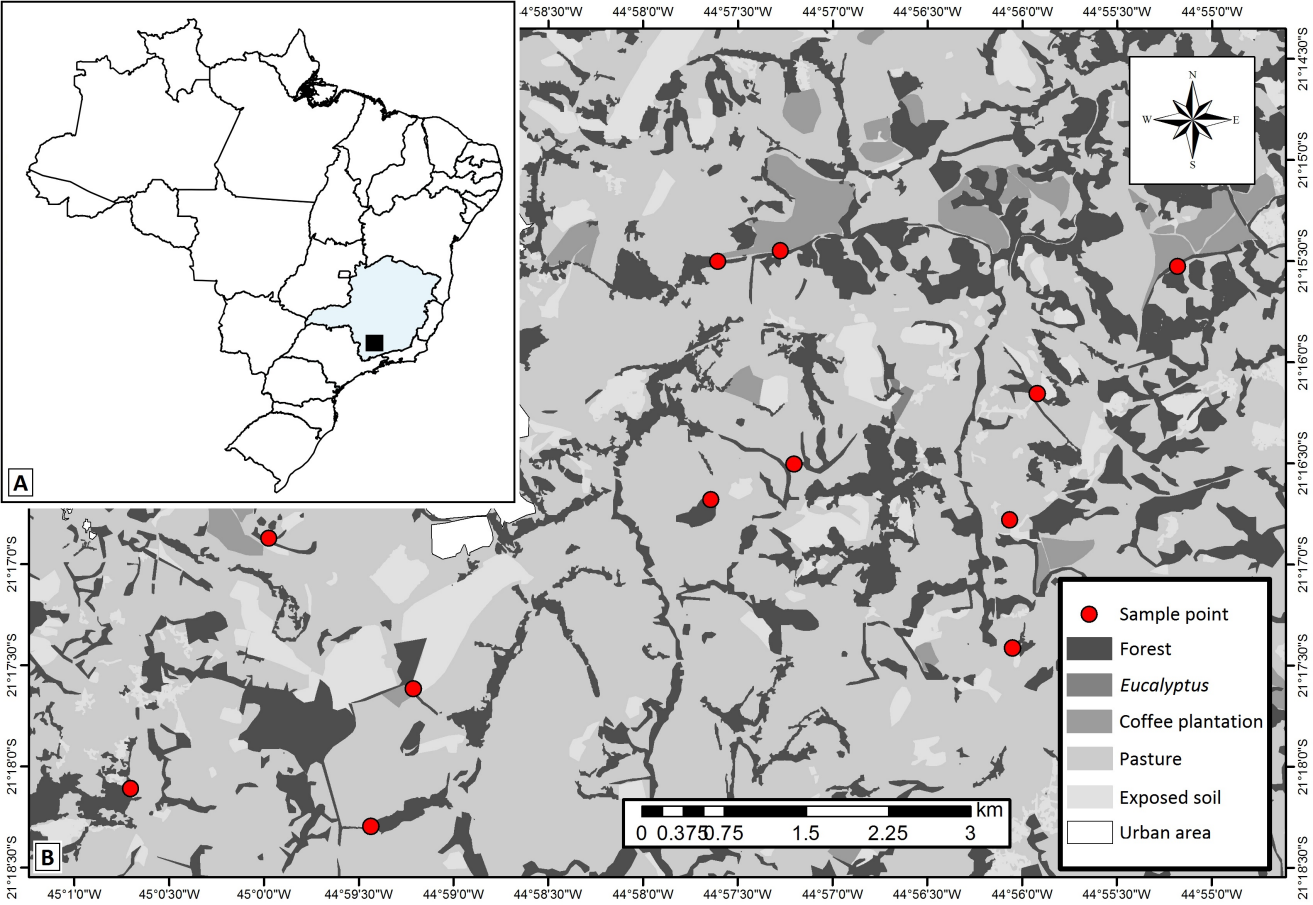
Study area map showing. (A) localization of the studied region within the Minas Gerais State—Brazil, (B) the 12 studied landscapes (represented by each sample point) and the different types of land use and cover classes in the studied region. Map thematic source: Tainá Assis.

The study was carried out in a 70-km^2^ area of the municipality of Lavras, southeastern Brazil (21°15′S–21°18′25″S; 45°00′57″W–44°54′34″W), in the transition between two biodiversity hotspots: the Cerrado (tropical savanna) and the Atlantic Forest (semideciduous seasonal forest) (Fig. 1). The climate in this region is humid subtropical (Cwa), according to Köppen climate classification, and experiences cold-dry winters and hot-rainy summers. The annual precipitation and mean temperature are 1,460 mm and 20.4 °C, respectively, and the elevation varies between 967 m and 1,055 m (Schiffler, 2003; Dantas, Carvalho & Ferreira, 2007). During the studied period (January 2011—summer) the total precipitation and mean temperature were about 1,364 mm and 23.0 °C, respectively (Source: INMET network data). This season is considered the best period of the year to sample dung beetles in tropical areas (Martínez & Vasquez, 1995; Lobo & Halffter, 2000; Milhomem, VazdeMello & Diniz, 2003).

The studied area has historically experienced pressures from agro-pastoral activities and urbanization, which generated the variegated landscapes. Overall, the landscapes are composed of fragments of secondary semideciduous forests (forest fragments) interconnected by hedgerows (forest corridors), and human settlements immersed in matrices of coffee plantations or introduced pastures. We delimited twelve 500 × 500 m experimental landscapes in which we selected one site of each of the main land uses: forest fragments (with average size of 18.25 ha), forest corridors (colonization of land plot boundary ditches, typical of this region—Castro & Van den Berg, 2013), coffee plantations (traditional management—*Coffea arabica* L.) and pastures grazed by cattle (exotic plants—*Urochloa* spp.). The selected forest corridors were connected to forest fragments and adjacent to coffee plantations and pastures. Coffee plantations were not present in four of the landscapes. Thus, we sampled 12 forest fragments, 12 forest corridors, 12 pastures and eight coffee plantations. We established one 150 m transect in each of the 44 sampling sites.

### Sampling of dung beetles

We sampled dung beetles using six pitfall traps, 30 m apart from each other, along each transect (Total: 6 × 44 = 264 traps). We used a smaller distance between traps than recommended by some authors (Larsen & Forsyth, 2005; Silva & Hernández, 2015) because our fragments were small and our sample unit was the area of LUCCs (each pitfall value of each dung beetle attribute was pooled in a sample unique per transect). Each pitfall trap consisted of a plastic container (19 cm diameter, 11 cm depth), half filled with a solution of saline and detergent (5%) to break the surface tension of the water and preserve dung beetles, and a hanging bait compartment with a lid to protect against rain and desiccation by the sun. We placed the pitfall traps, which were baited with 30 g of homogenized human feces, between 9:00 am and 4:00 pm, and left them open for a 48 h period. After sampling, dung beetles were sorted, counted and identified to the lowest taxonomic level possible with the help of available taxonomic keys (e.g., Vaz-de-Mello et al., 2011) and the CREN (Neotropical Dung Beetles Reference Collection, at the Universidade Federal of Lavras). Voucher specimens were deposited at CREN. All dung beetles were dried at 40 °C in order to preserve the specimens and to obtain their dry body weight. In order to calculate species mean biomass, we weighed 20 individuals (or the maximum possible) of each species using a precision scale balance (0.0001 g).

Dung beetles were sampled on farms with the permission of the landholders. We also possessed an IBAMA/SISBIO license (number 10061-1) in the name of Julio Louzada. In addition, the feces used in the study was donated by the authors, who all agreed on donating it.

### Measuring structural differences among land classes

To explain variation in the studied community parameters, we measured three environmental variables reflecting structural differences among LUCCs: canopy cover (CC), local vegetation heterogeneity (LH) and soil sand content (SS). To estimate CC, we used hemispherical canopy photographs taken 1.5 m above the soil next to each pitfall trap with an 8-mm fisheye lens (Engelbrecht & Herz, 2001). We analyzed the photographs using the software Gap Light Analyzer 2.0 (GLA, Frazer, Canham & Lertzman, 1999) and quantified the percentage of pixels related to vegetation in each photograph as a proxy for canopy cover. We measured the fractal dimension (number that characterizes the geometry of a fractal) of the understory vegetation to use as a proxy for LH. To do so, we took photographs of the understory, according to a methodology adapted from Nobis (2005), and analyzed them in the software SIDELOOK (Nobis, 2005), which calculates the fractal dimension of each photograph. Photographs of a black panel (1 × 1 m) placed behind vegetation 3 m away were taken with a camera with a 52-mm lens positioned 1 m above the soil adjacent to each pitfall trap. To measure SS, we used a homogenization of all the soil samples taken next to the pitfall traps of a transect (Total = 44 samples). Homogenized soil samples were analyzed for their texture, meaning content of sand, silt and clay in each soil sample. As these variables are highly correlated, we only used sand content (percentage in the sample), as a measurement of soil structure. Sand content is a soil variable related to an important dung beetle behavior (digging) that plays an essential role in ecosystem functioning (Halffter & Edmonds, 1982; Davis, 1996; Griffiths et al., 2015).

### Data analysis

Comparisons of species richness among LUCCs could be biased because of possible differences in sample coverage or low sample coverage—which would mean that dung beetle communities were under-sampled (Chao & Jost, 2012). To make more accurate comparisons, we calculated LUCC-level sampling coverage using iNEXT package in R (Chao et al., 2014; Hsieh, Ma & Chao, 2016). This package also allows us to compare species richness of standardized samples at the same sample completeness based on a rarefaction/extrapolation sampling curve (R/E curve) (Hsieh et al., 2016).

We used dung beetle species richness, abundance, biomass, and community structure as response variables and LUCC as the explanatory variable to answer our first question. First, we used Generalized Linear Models (GLM) with species richness (total number of species per transect), abundance (total number of individuals per transect) and biomass (total dry body weight per transect) as response variables. We submitted models to pairwise contrast analysis (lsmeans package—Lenth, 2016), in order to combine statistically similar classes of land uses and cover. Models were built and compared using R language (R Development Core Team, 2015). Second, we conducted Principal Coordinate Analysis (PCO—Gower, 1966) followed by Permutational Multivariate Analysis of Variance (PERMANOVA—McArdle & Anderson, 2001)—to test for significant clustering of sites with respect to different LUCCs. We used community structure (matrix based on square-root transformed abundance data and Bray Curtis dissimilarity index) as the response variable. Finally, we performed tests for homogeneity of multivariate dispersions (PERMDISP—Anderson, 2006), to check for differences in variance dispersion of community structure data among LUCCs. This analysis was performed using the software Primer v.6 with PERMANOVA + (Clarke & Gorley, 2006).

We used dung beetle community structure as the response variable and CC, LH and SS as explanatory variables to answer our second question. First, we used Hierarchical Partitioning to assess the influence of CC, LH and SS on species richness, abundance and biomass. This method provides an estimate of the independent effects of each explanatory variable on the response variable (Chevan & Sutherland, 1991; Mac Nally, 2000). We performed this analysis using R language (R Development Core Team , 2015). Second, we used Distance-based Multivariate Analysis for a Linear Model (DistLM, Legendre & Anderson, 1999; McArdle & Anderson, 2001) to assess the influence of CC, LH and SS on community structure. DistLM analyzes and models the relationship between a multivariate data cloud and one or more independent variables (Anderson, Gorley & Clarke, 2008). DistLM allows independent variables to be fitted individually or together in user specified sets. The DistLM routine was based on the AICc model selection criterion (Burnham & Anderson, 2004) using a “step-wise” selection procedure. Primer 6.0 and PERMANOVA+ for PRIMER software were used (Clarke & Gorley, 2006; Anderson, Gorley & Clarke, 2008).

In order to answer our third question, we decomposed beta diversity of dung beetle communities into spatial turnover and nestedness-resultant components to determine their relative contributions to beta-diversity patterns in the studied landscapes. The beta diversity was decomposed into Sørensen (β_SOR_) and Simpson (β_SIM_) dissimilarity indices (Baselga, 2010). Sørensen (β_SOR_) dissimilarity represents the total beta diversity and incorporates both species replacement and nestedness-resultant dissimilarities. Simpson (β_SIM_) dissimilarity describes species turnover, or replacement, and it is equal to β_SOR_ in the absence of nestedness. Thus, the difference between these indices is a measure of the nestedness-resultant component of beta diversity (β_SNE_ = β_SOR_ − β_SIM_ (Baselga, 2010)).

We calculated multiple-site dissimilarity to estimate the overall beta diversity of dung beetle communities among all sites in each landscape (Baselga, 2013). In order to represent the relative contribution of the nestedness-resultant component to overall beta diversity, we calculated its proportion for overall multiple-site dissimilarity (βratio =β_SNE_∕β_SOR_). Where, βratio <0.5 represents dominance of species replacement in beta diversity patterns and βratio >0.5 represents dominance of the nestedness-resultant component (Dobrovolski et al., 2012).

**Figure 2:**
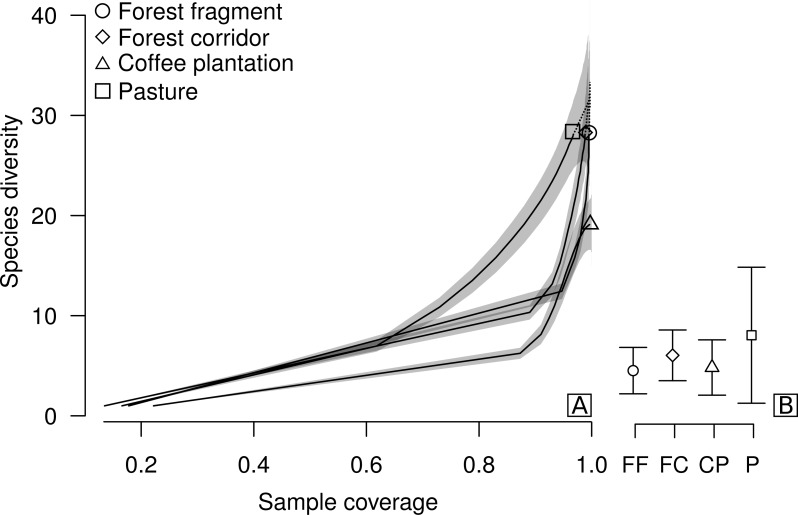
Sample coverage-based species accumulation curve of dung beetle sampled in forest fragment, forest corridor, coffee plantation, and pasture of 12 landscapes in Lavras, Brazil (A). Estimated average species richness and standard deviation at the same sample coverage (77.6%) in FF, forest fragment; FC, forest corridor; CP, coffee plantation and P, pasture (B). The shaded area indicates the 95% confidence interval and the dashed line represents extrapolation data.

## Results

We collected a total of 2,695 individuals of 52 species of dung beetles from the tribes Ateuchini (three genera, 11 species), Delthochilini (five genera, 13 species), Coprini (five genera, 14 species), Oniticellini (one genus, four species), Onthophagini (one genus, two species) and Phanaeini (four genera, eight species). Of these, 28 species occurred in forest fragments (1,549 individuals), forest corridors (603 individuals) and pastures (211 individuals), and 19 species in coffee plantations (332 individuals) (Table 1). The highest average sample coverage of our sampled LUCCs was for forest fragment samples (SC = 93.8%) and the lowest coverage was in pasture samples (SC = 77.6%—coffee plantation = 92.49%, and forest corridor = 85.56%) (Table S1). When all LUCCs were compared at equal sample coverage (in this case, we used rarefied coverages at the lowest average value—app. 77.6%), estimated average species richness showed a different trend than those of the raw data. All LUCCs had the same estimated species richness (Fig. 2B; Table S2). Our R/E coverage-based curves (based on pooled data) showed similar patterns of species accumulation between forest fragments and forest corridors (Fig. 2A).

**Table 1:** Dung beetles collected at Forest fragments (FF), Forest corridors (FC), Coffee plantation (CP), and Pasture (P) in Lavras—Brazil.

**Tribe/Species**	**FF**	**FC**	**CP**	**P**	**Biome**
**Ateuchini**					
*Ateuchus* aff. *carbonarius* (Harold, 1868)	0	1	0	0	–
*Ateuchus* sp.	6	1	0	0	–
*Ateuchus striatulus* (Borre, 1886)	0	0	0	2	C
*Canthidium* aff. *sulcatum* (Perty, 1830)	0	0	2	0	–
*Canthidium aterrimum* Harold, 1867	403	28	125	19	AF
*Canthidium barbacenicum* Borre, 1886	0	3	2	4	C
*Canthidium decoratum* (Perty, 1830)	0	0	0	5	C
*Canthidium* sp.1	0	1	2	0	–
*Canthidium* sp.2	0	0	0	1	–
*Canthidium* sp.3	2	0	0	0	–
*Uroxys* sp.	1	3	14	1	–
**Delthochilini**					
*Canthon* sp.1	13	67	0	0	–
*Canthon* aff. *podagricus* Harold, 1868	0	0	0	10	–
*Canthon chalybaeus* Blanchard, 1843	0	2	48	0	AF
*Canthon lituratus* (Germar, 1824)	0	0	0	2	C
*Canthon septemmaculatus histrio* (Serville, 1828)	18	0	1	0	C
*Canthon* sp.2	1	1	0	0	–
*Canthon virens* Mannerheim, 1829	0	0	0	5	C
*Deltochilum orbignyi* (Blanchard, 1845)	0	0	0	1	–
*Deltochilum rubripenne* Gory, 1831	81	1	0	0	–
*Deltochilum* sp.	9	4	0	0	–
*Pseudocanthon* aff. *xanthurus* (Blanchard, 1843)	0	0	0	5	–
*Scybalocanthon korasakiae* Silva, 2011	219	96	0	0	AF
*Sylvicanthon foveiventris* Schmidt, 1920	390	95	0	0	AF
**Coprini**					
*Dichotomius* aff. *rotundigena* Felsche, 1901	1	3	2	0	–
*Dichotomius affinis* Felsche, 1910	26	4	0	2	–
*Dichotomius bicuspis* (Germar, 1824)	22	34	68	1	AF
*Dichotomius bos* (Blanchard, 1843)	2	2	4	78	C
*Dichotomius carbonarius* Mannerheim, 1829	5	52	44	7	C/AF
*Dichotomius depressicollis* (Harold, 1867)	4	1	0	0	AF
*Dichotomius fissus* Harold, 1867	5	0	0	0	AF
*Dichotomius mormon* Ljungh, 1799	234	29	2	1	AF
*Dichotomius nisus* (Olivier, 1789)	0	0	0	2	C
*Dichotomius* sp.	0	2	0	0	–
*Eutrichillum hirsutum* Boucomont, 1928	0	0	0	3	C
*Isocopris inhiatus* (Germar, 1824)	0	0	0	2	–
*Ontherus azteca* Harold, 1869	5	4	0	0	C/AF
*Trichillum externepunctatum* Borre, 1886	1	0	0	6	C
**Oniticellini**					
*Eurysternus caribaeus* (Herbst, 1789)	10	9	0	0	AF
*Eurysternus cyanescens* Balthasar, 1939	1	0	0	0	C/AF
*Eurysternus hirtellus* Dalman, 1824	38	6	0	0	AF
*Eurysternus parallelus* Castelnau, 1840	44	146	1	1	C/AF
**Onthophagini**					
*Onthophagus* aff. *hircullus* Mannerheim, 1829	0	0	2	0	–
*Onthophagus ranunculus* Arrow, 1913	0	0	2	25	C
**Phanaeini**					
*Coprophanaeus cyanescens* Olsoufieff, 1924	1	5	4	4	C/AF
*Coprophanaeus horus* Waterhouse, 1891	0	0	5	11	C
*Coprophanaeus spitzi* (Pessôa 1935)	0	0	2	4	C
*Dendropaemon* sp.	1	1	0	0	–
*Oxysternon palaemon* (Laporte, 1840)	0	0	0	6	C
*Phanaeus kirbyi* Vigors, 1825	0	0	0	1	C
*Phanaeus palaeno* Blanchard, 1843	0	0	2	2	C
*Phanaeus splendidulus* Fabricius, 1781	6	2	0	0	AF
**Abundance**	**1,549**	**603**	**332**	**211**	**–**
**Species Richness**	**28**	**28**	**19**	**28**	**–**

**Notes.**

AF species registered in Atlantic Forest samples C species registered in *Cerrado* samples “–” uncertain/without identification, based on Almeida et al. (2011), Campos & Hernández (2013) and Audino, Louzada & Comita (2014), Costa et al., 2016, unpublished data

**Figure 3:**
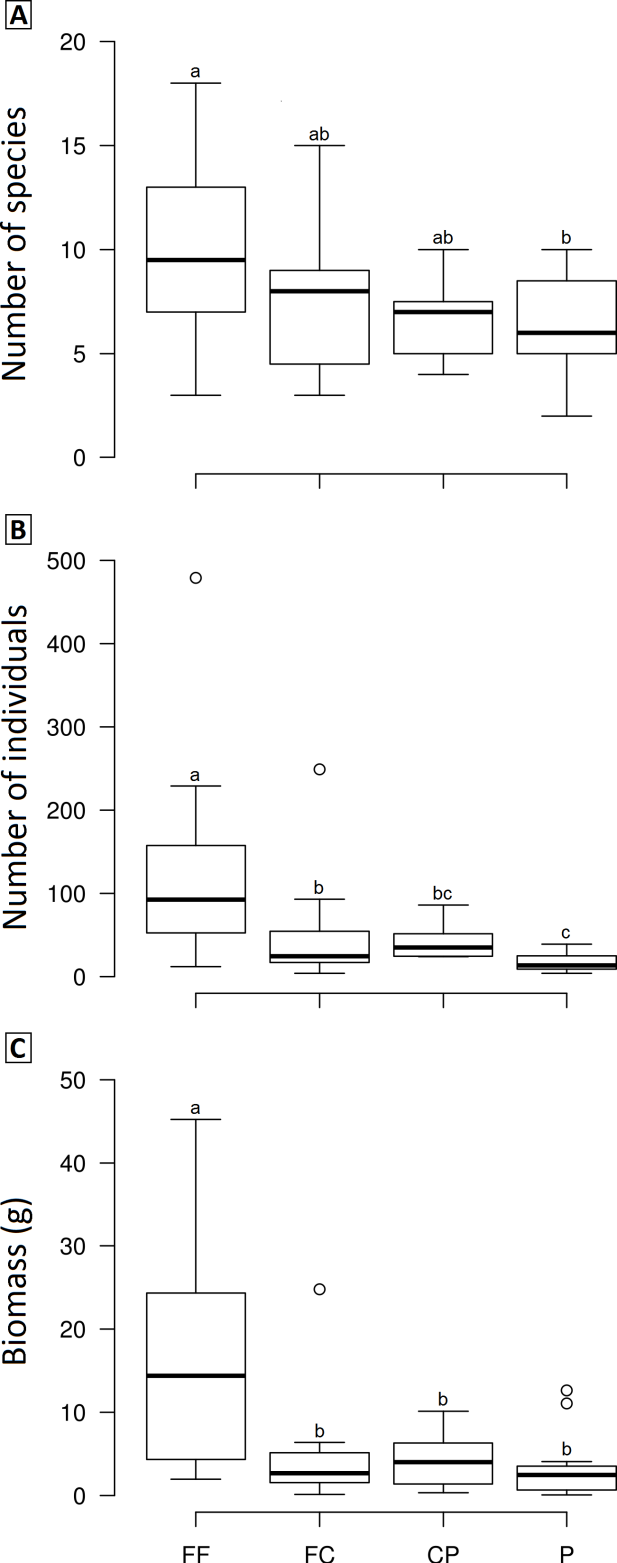
Boxplots showing the richness (A), abundance (B) and biomass (C) of dung beetle across the land use and cover classes in Lavras—Brazil. FF, forest fragment; FC, forest corridor; CP, coffee plantation and P, pasture. Different letters means significant differences at *p* < 0.05 among the land uses and cover classes.

We observed significant differences in the mean species richness, abundance and biomass among LUCCs (*F*_richness_ = 2.8978, *p* = 0.04, *df* = 3; *F*_abundance_ = 6.7067, *p* < 0.001, *df* = 3; *F*_biomass_ = 7.1122, *p* < 0.001, *df* = 3). Overall, forest fragments exhibited higher values than the other LUCCs, while forest corridors and coffee plantations had intermediate values, and pastures the lowest values; however, some pair-to-pair comparisons were not significantly different (Fig. 3). All pair-to-pair comparisons and results can be accessed in Table S3.

**Figure 4:**
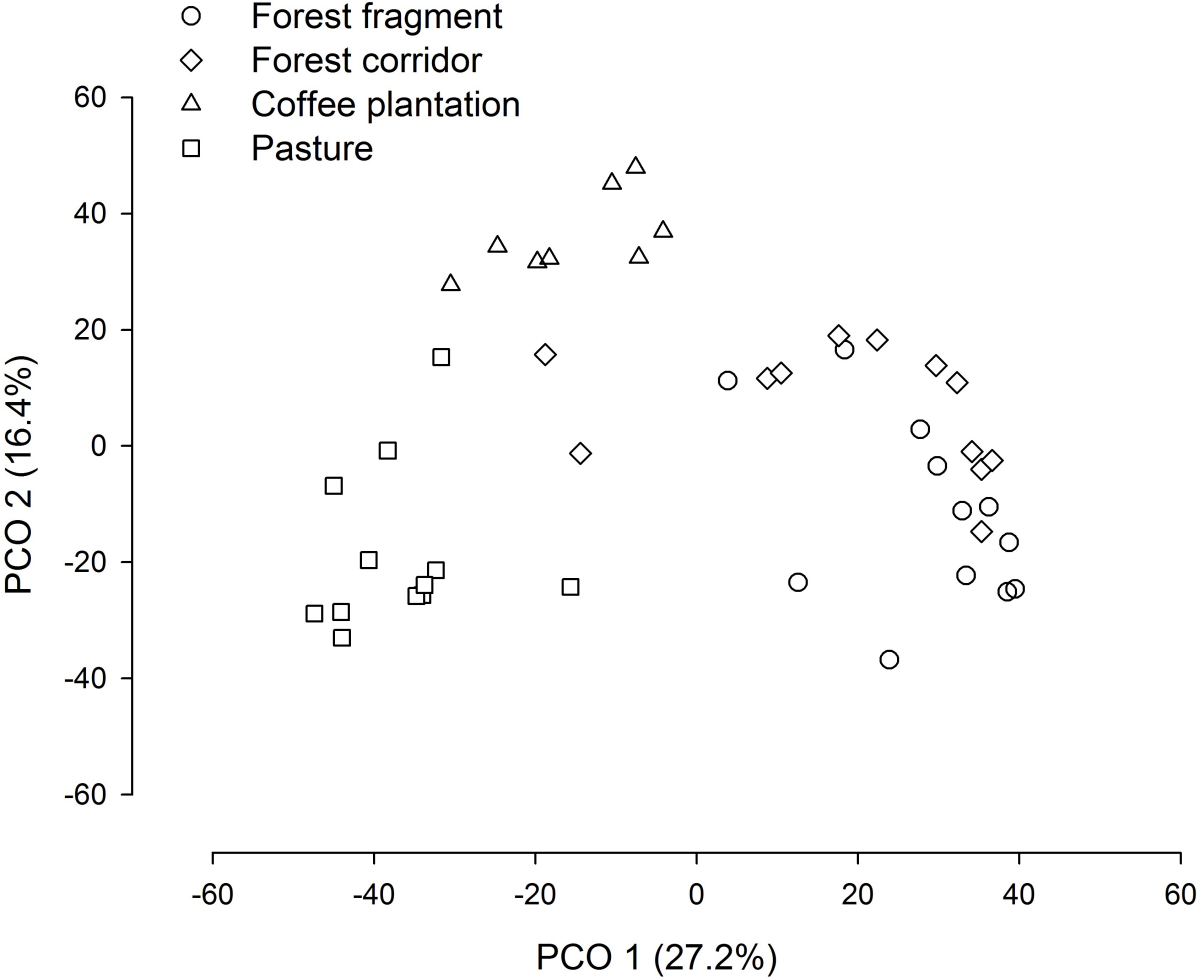
PCO biplot of Bray–Curtis similarity matrix based on square-root transformed dung beetle abundance data in land use and cover classes.

The PCO revealed three distinct groups (forest fragment + forest corridor, coffee plantation, and pasture) with axis 1 and 2 explaining 43.6% of the variation in structure (species composition—Fig. 4). However, dung beetle community structure was significantly different among the LUCCs (PERMANOVA, pseudo-F = 8.0969, *p* = 0.001, *df* = 3) (Table S4). The LUCCs also exhibited differences in the dispersion of the variance of the community structure data (PERMDISP, *F* = 3.5964, *p* = 0.05, *df* = 3), with higher values in pasture in comparison to forest fragment (*t* = 2.9631, *p* = 0.017) and coffee plantation (*t* = 4.1819, *p* = 0.003) (Table S4).

**Table 2:** Results of hierarchical partitioning analyses with all the environmental variables.

**Variables**	***%I***	***I***	***J***	*I* + *J* = *R*^2^	$\sqrt{I}$
(a) Total richness					
CC	83.072^*^	0.127	0.009	0.14	+0.37
SS	5.375	0.008	−0.003	0.005	0.09
LH	11.553	0.018	0.012	0.03	0.13
(b) Total abundance					
CC	80.101^*^	0.130	0.031	0.162	+0.36
SS	0.424	0.0007	−0.0007	0.00003	0.03
LH	19.475	0.032	0.032	0.063	0.18
(c) Total biomass					
CC	83.047^*^	0.13	0.026	0.156	+0.4
SS	0.537	0.0008	0.0006	0.001	0.03
LH	16.415	0.026	0.025	0.050	0.2

**Notes.**

*%I* percentage of independent effect *I* independent explanatory power of the variable *J* Joint explanatory power of the variable with all other variables listed ( *I* + *J* = *R*^2^) univariate squared correlation, and $\sqrt{(I)}$ square root of the independent explanatory power, which may be interpreted as the independent correlation with the response variable; the sign is allocated to that of the univariate correlation CC canopy cover SS soil sand content LH local vegetation heterogeneity

* Indicates statistically significant variables at *p* ≤ 0.05.

Canopy cover (CC) significantly influenced all community parameters studied. Hierarchical partitioning revealed positive effects of CC on dung beetle species richness (83.07% of independent effect), abundance (80.1%) and biomass (83.04%) (Table 2). Likewise, community structure exhibited the same pattern (22.35% of independent effect) (Pseudo-F = 12.090, *p* < 0.001, *df* = 42) (Table 3).

**Figure 5:**
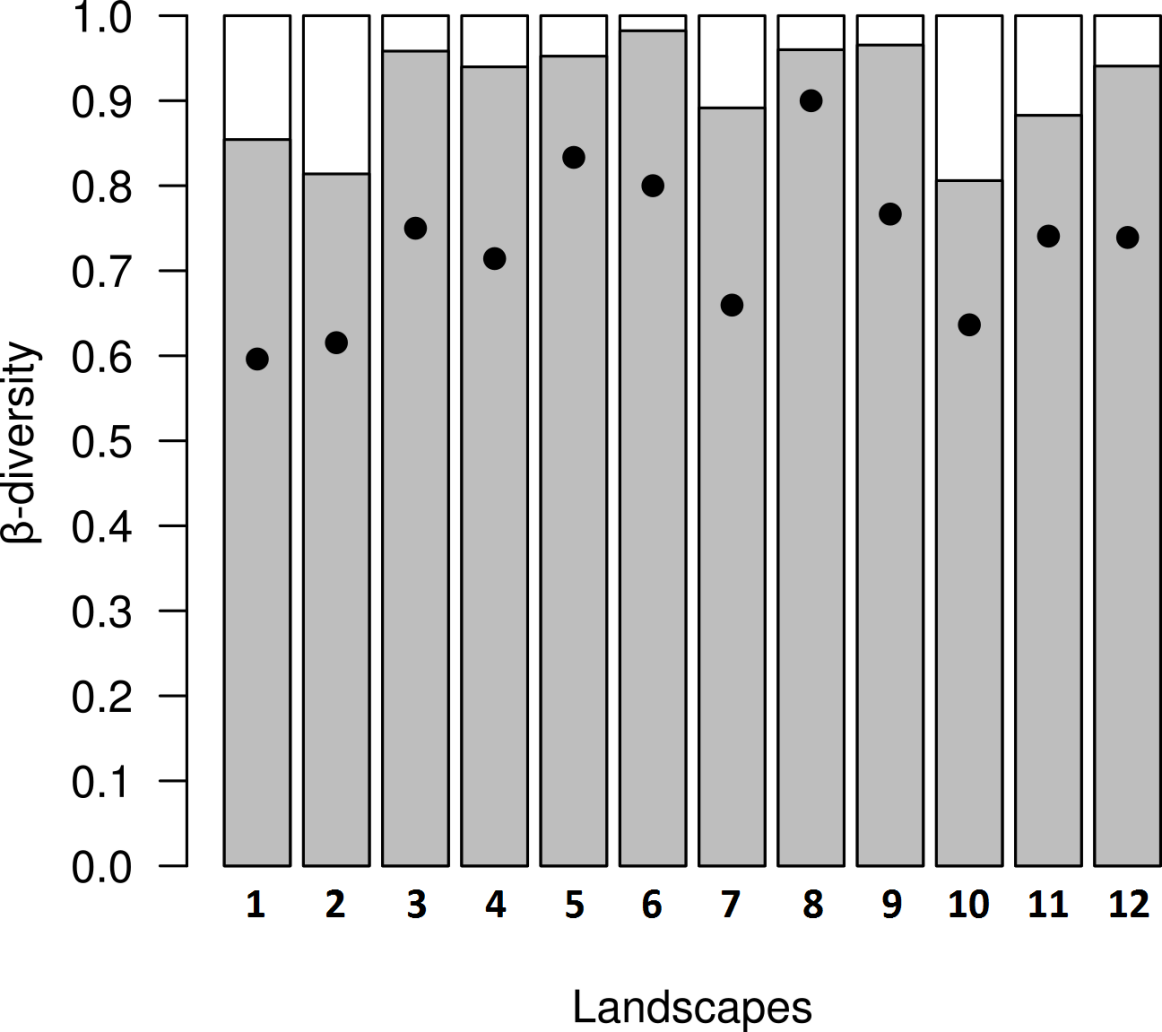
Results from decomposition of dung beetle beta diversity in four land use and cover classes at 12 variegated landscapes in Lavras— MG, Brazil. Grey bars represent spatial turnover component proportion (β_SIM_∕β_SOR_), white bars represent nestedness-resultant component proportion (β_SNE_∕β_SOR_), and black dots represent overall values of beta diversity (β_SNE_ + β_SIM_) in each landscape.

**Table 3:** Results of distance based linear models (DistLM). Response variable is dung beetle species composition and predictor variables are canopy cover (CC), soil sand content (SS), and local vegetation heterogeneity (LH).

**Variable**	**AICc**	**SS**(trace)	**Pseudo-F**	**P**	**Prop**	**Cumulative**	**res.df**
**Marginal tests**							
SS	–	3555.1	1.071	0.3621	0.025	–	42
CC	–	31,958	12.1	0.0001	0.2235	–	42
LH	–	18,590	6.3	0.0001	0.130	–	42
**Sequential tests**							
CC	348.96	31,958	12.1	0.0001	0.2235	0.2235	42

**Notes.**

Prop Proportion of explained variation

In the variegated landscapes studied, the decomposition of beta diversity revealed that the main process driving beta diversity in these landscapes was spatial turnover, with βratio <0.5 in all landscapes (Fig. 5).

## Discussion

This study evaluated the ecological patterns of dung beetle communities in variegated tropical landscapes, highlighting the importance of these landscapes for conservation of tropical biodiversity. Our 12 studied landscapes presented diverse dung beetle communities that were structurally different among the LUCCs (high beta diversity), and capable of sustaining several species from both the Atlantic Forest and Cerrado (mainly from open physiognomies).

Dung beetle communities respond differently to the LUCCs present in our studied landscapes, with variation in species richness, abundance, biomass, and community structure. The presence of well-defined communities in each LUCC highlights the importance of their maintenance for conserving regional diversity. Because LUCCs vary regarding their permeability to forest (fragments, corridors, and coffee) and Cerrado (coffee and pasture) species, the studied landscapes were able to sustain a significant group of native species from both the Atlantic Forest and Cerrado found in this region (Table 1) (Schiffler, 2003; Almeida & Louzada, 2009; Almeida et al., 2011; Barragán et al., 2011; Gries et al., 2012). However, in each modified LUCC (pasture, coffee plantation, and forest corridor) we found more species at the regional level (all landscapes) than at the local scale (per landscape). The low dung beetle abundance and biomass observed lead us to believe that these land use classes are used as transitional habitats for dung beetles, such as ecological corridors or stepping-stones (Fagan, Cantrell & Cosner, 1999; Estrada & Coates-Estrada, 2002; Fischer & Lindenmayer, 2007; Díaz, Galante & Favila, 2010; Almeida et al., 2011).

These land use classes also harbor exclusive species, as the main process promoting beta diversity in these landscapes is spatial replacement. In consolidated landscapes, such as studied in this paper, environmental filters have already acted in each of the studied LUCCs, showing that some dung beetle species apparently recognize different LUCCs as habitats (Webb et al., 2010). The ability of a species to survive in human-modified landscapes is of great importance (Gardner et al., 2009), since currently most ecosystems suffer some level of perturbation. Of the environmental factors measured in this study, the most important was canopy cover. This variable is often reported in scientific literature as a proxy for habitat quality and resource availability for dung beetles (Halffter & Matthews, 1966; Halffter, 1991; Halffter & Arellano, 2002; Louzada et al., 2010; Audino, Louzada & Comita, 2014). Although soil and vegetation parameters can influence dung beetle communities (Gries et al., 2012; Farias et al., 2015), because they can affect larvae survival (Osberg, Doube & Hanrahan, 1994; Davis et al., 2010), the present work found LH and SS not liable for determining dung beetle community structure.

Our results provide additional evidence that variegation of a landscape can allow species movement between habitats of variable suitability, favoring their long-term persistence (Doerr et al., 2014). Such a scenario offers a better outlook for biodiversity conservation than scenarios resulting from fragmentation. Fragmentation tends to confine species in reduced patches of low-quality habitat, eventually leading populations to suffer from problems related to endogamy (e.g., reduced genetic variability; enhanced susceptibility to diseases and stochastic events) and local extinctions (Keller & Largiadèr, 2003; Keyghobadia, 2007; Delaney, Riley & Fisher, 2010). In our studied landscapes, we recorded several species typical of the Atlantic Forest in forest fragments, forest corridors and coffee plantations (Table 1). In addition, Cerrado species were dominant in pastures (Table 1), suggesting that local species may be able to persist in human modified landscapes if enough time is given, and if introduced habitats conserve at least some structural similarity with natural ones.

Based on the ability of our studied modified habitats to conserve native species and contribute to increased regional diversity, we encourage the consideration of variegation of previously fragmented landscapes in the management of human modified landscapes (Rös, Escobar & Halffter, 2012). Diversification of LUCCs may benefit biodiversity, improve regional heterogeneity and connectivity, and help maintain the provisioning of critical ecological functions (Andresen, 2002; Nichols et al., 2008; Braga et al., 2012; Braga et al., 2013). In transitional areas, this diversification could be even more important for species conservation. Human modification generally favors the occurrence of open landscapes by: (a) suppressing the natural habitat of species from at least two habitat types (e.g., in a forest-forest transition), (b) favoring species of a single habitat type (e.g., forest to non-forest transition), (c) homogenizing two open habitat types (e.g., conversion of native savannas and fields into pastures) or (d) suppressing at least two natural habitat types (e.g., urbanization).

Finally, we caution against ignoring the negative effects of deforestation and habitat degradation. For instance, a recent study by Barlow et al. (2016) showed that human disturbances in the Amazon forest were responsible for reducing the diversity of dung beetles, birds and plants with almost two times the strength of deforestation. This reinforces the irreplaceability of natural habitats for biodiversity conservation and highlights the need to reduce disturbances in the remaining habitats. Our results reflect a 300-year old scenario of human-induced modification, which despite showing relatively good prospects for biodiversity conservation, was responsible for the suppression of large areas of natural habitats. Therefore, our studied landscapes may have already experienced strong biodiversity losses. In face of current rates of tropical deforestation, degradation and land use intensification, such landscapes are becoming more common. We encourage the variegation of landscapes as a complementary initiative of current conservation practices (e.g., protection of natural habitats and establishment of reserves). Together, these management strategies may achieve partial recovery of biodiversity and ecosystem functioning, even when landscapes are not able to sustain the entire biodiversity of native communities (Barlow et al., 2010; Gray et al., 2016).

##  Supplemental Information

10.7717/peerj.3125/supp-1 Table S1 Sample coverage Sample coverage and observed richness of all land use and cover classes (LUCC) in the twelve variegated landscapes of Lavras —MG, Brazil.

10.7717/peerj.3125/supp-2 Table S2 Estimated Species Richness Estimated richness of all land use and cover classes (LUCC) at equal average sample coverage (app. 77.6%) in the twelve variegated landscapes of Lavras —MG, Brazil.

10.7717/peerj.3125/supp-3 Table S3 Contrast analysis Paired test values of contrast analysis to assess differences in species richness, abundance, and biomass of dung beetle among the land use and cover classes (forest fragments, forest corridors, coffee plantation and pasture), Lavras —Brazil. ∗ highlights significant results at *p* < 0.05.

10.7717/peerj.3125/supp-4 Table S4 PERMANOVA and PERMDISP analysis Paired test values of PERMANOVA and PERMDISP analysis to assess, respectively, differences in species composition and data cloud multivariate dispersion among the land use and cover classes (forest fragments, forest corridors, coffee plantation and pasture), Lavras —Brazil. The mean values of multivariate dispersion were fragment = 39.34 (±3.24), corridor = 44.79 (±4.46), coffee = 35.46 (±3.15), and pasture = 50.88 (±2.17)—df1 = 3 and df2 = 40.

10.7717/peerj.3125/supp-5 Data S1 Raw data

## Additional Information and Declarations

### Field Study Permissions

The following information was supplied relating to field study approvals (i.e., approving body and any reference numbers): The MMA/IBAMA/SISBIO approved this work under licence number 10061-1.

### Data Availability

The following information was supplied regarding data availability: The raw data has been supplied as Supplementary File.
