# Supplementary material for: Variegated tropical landscapes conserve diverse dung beetle communities
Source: PeerJ. 2017 Apr 4;5:e3125. doi: 10.7717/peerj.3125 (PMC5382926; doi:10.7717/peerj.3125)
Supplement: Table S1 — Sample coverage and observed richness of all land use and cover classes (LUCC) in the twelve variegated landscapes of Lavras —MG, Brazil. [file peerj-05-3125-s001.doc]

Table S1 Sample coverage and observed richness of all land use and cover classes (LUCC) in the twelve variegated landscapes of Lavras – MG, Brazil.

| **LUCC** | **Site** | **N** | **Sample Coverage** | **Observed Richness** | **Singletons** | **Doubletons** |
| --- | --- | --- | --- | --- | --- | --- |
| Forest Fragment | 1 | 479 | 0.9854 | 18 | 7 | 0 |
| Forest Fragment | 2 | 162 | 0.9941 | 11 | 1 | 4 |
| Forest Fragment | 3 | 68 | 0.9857 | 6 | 1 | 1 |
| Forest Fragment | 4 | 105 | 0.9718 | 12 | 3 | 2 |
| Forest Fragment | 5 | 12 | 0.598 | 7 | 5 | 1 |
| Forest Fragment | 6 | 65 | 0.9389 | 9 | 4 | 1 |
| Forest Fragment | 7 | 80 | 0.9262 | 15 | 6 | 4 |
| Forest Fragment | 8 | 17 | 1 | 3 | 0 | 1 |
| Forest Fragment | 9 | 229 | 0.9826 | 7 | 4 | 1 |
| Forest Fragment | 10 | 139 | 0.9857 | 10 | 2 | 1 |
| Forest Fragment | 11 | 153 | 0.9609 | 14 | 6 | 1 |
| Forest Fragment | 12 | 40 | 0.9263 | 7 | 3 | 1 |
| **Average** | **-** | **129.1** | **0.9379** | **9.92** | **3.5** | **1.5** |
| Corridor Strip | 1 | 249 | 0.984 | 15 | 4 | 0 |
| Corridor Strip | 2 | 19 | 0.8006 | 9 | 4 | 2 |
| Corridor Strip | 3 | 19 | 0.748 | 9 | 5 | 2 |
| Corridor Strip | 4 | 93 | 0.968 | 9 | 3 | 1 |
| Corridor Strip | 5 | 4 | 0.625 | 3 | 2 | 1 |
| Corridor Strip | 6 | 40 | 0.9524 | 8 | 2 | 2 |
| Corridor Strip | 7 | 62 | 0.9531 | 12 | 3 | 3 |
| Corridor Strip | 8 | 20 | 1 | 4 | 1 | 0 |
| Corridor Strip | 9 | 6 | 0.3939 | 5 | 4 | 1 |
| Corridor Strip | 10 | 47 | 1 | 7 | 1 | 0 |
| Corridor Strip | 11 | 29 | 0.9013 | 8 | 3 | 2 |
| Corridor Strip | 12 | 15 | 0.9417 | 4 | 1 | 1 |
| **Average** | **-** | **50.25** | **0.8556** | **7.75** | **2.75** | **1.25** |
| Coffee Plantation | 1 | 45 | 0.9343 | 8 | 3 | 1 |
| Coffee Plantation | 2 | 26 | 0.969 | 6 | 1 | 3 |
| Coffee Plantation | 4 | 86 | 0.9655 | 7 | 3 | 0 |
| Coffee Plantation | 6 | 24 | 0.7952 | 7 | 5 | 1 |
| Coffee Plantation | 7 | 25 | 0.8464 | 10 | 4 | 2 |
| Coffee Plantation | 9 | 58 | 0.9661 | 7 | 2 | 1 |
| Coffee Plantation | 11 | 44 | 1 | 4 | 0 | 2 |
| Coffee Plantation | 12 | 24 | 0.9233 | 4 | 2 | 0 |
| **Average** | **-** | **41.5** | **0.9249** | **6.625** | **2.5** | **1.25** |
| Pasture | 1 | 37 | 0.8393 | 10 | 6 | 1 |
| Pasture | 2 | 4 | 1 | 2 | 1 | 0 |
| Pasture | 3 | 20 | 0.9174 | 8 | 2 | 4 |
| Pasture | 4 | 9 | 0.249 | 8 | 7 | 1 |
| Pasture | 5 | 10 | 0.6276 | 6 | 4 | 0 |
| Pasture | 6 | 30 | 0.9065 | 9 | 3 | 3 |
| Pasture | 7 | 9 | 0.7333 | 6 | 3 | 3 |
| Pasture | 8 | 14 | 0.5125 | 9 | 7 | 0 |
| Pasture | 9 | 18 | 0.8426 | 5 | 3 | 0 |
| Pasture | 10 | 8 | 1 | 2 | 1 | 0 |
| Pasture | 11 | 13 | 0.7814 | 5 | 3 | 1 |
| Pasture | 12 | 39 | 0.8992 | 5 | 4 | 0 |
| **Average** | **-** | **17.58** | **0.7757** | **6.25** | **3.67** | **1.084** |
