# Supplementary material for: Variegated tropical landscapes conserve diverse dung beetle communities
Source: PeerJ. 2017 Apr 4;5:e3125. doi: 10.7717/peerj.3125 (PMC5382926; doi:10.7717/peerj.3125)
Supplement: Table S2 — Estimated richness of all land use and cover classes (LUCC) at equal average sample coverage (app. 77.6%) in the twelve variegated landscapes of Lavras —MG, Brazil. [file peerj-05-3125-s002.doc]

Table S2 Estimated richness of all land use and cover classes (LUCC) at equal average sample coverage (app. 77.6%) in the twelve variegated landscapes of Lavras – MG, Brazil.

| **LUCC** | **Site** | **Method** | **N** | **Sample Coverage** | **Estimated Richness** |
| --- | --- | --- | --- | --- | --- |
| Forest Fragment | 1 | rarefaction | 8 | 0.7812 | 3.978 |
| Forest Fragment | 2 | rarefaction | 5 | 0.7769 | 2.389 |
| Forest Fragment | 3 | rarefaction | 5 | 0.7876 | 2.694 |
| Forest Fragment | 4 | rarefaction | 10 | 0.7722 | 4.869 |
| Forest Fragment | 5 | extrapolation | 28 | 0.773 | 11.988 |
| Forest Fragment | 6 | rarefaction | 7 | 0.7631 | 3.882 |
| Forest Fragment | 7 | rarefaction | 14 | 0.776 | 6.154 |
| Forest Fragment | 8 | rarefaction | 3 | 0.7771 | 1.781 |
| Forest Fragment | 9 | rarefaction | 2 | 0.7971 | 1.378 |
| Forest Fragment | 10 | rarefaction | 8 | 0.7704 | 3.35 |
| Forest Fragment | 11 | rarefaction | 9 | 0.777 | 4.636 |
| Forest Fragment | 12 | rarefaction | 7 | 0.7795 | 2.848 |
| **Average** | **-** | **-** | **8.83** | **0.7775** | **4.2** |
| Corridor Strip | 1 | rarefaction | 11 | 0.7761 | 5.127 |
| Corridor Strip | 2 | rarefaction | 17 | 0.7778 | 8.567 |
| Corridor Strip | 3 | extrapolation | 22 | 0.7789 | 9.724 |
| Corridor Strip | 4 | rarefaction | 5 | 0.7806 | 2.707 |
| Corridor Strip | 5 | extrapolation | 6 | 0.7891 | 3.656 |
| Corridor Strip | 6 | rarefaction | 10 | 0.7784 | 4.621 |
| Corridor Strip | 7 | rarefaction | 14 | 0.7752 | 6.934 |
| Corridor Strip | 8 | rarefaction | 4 | 0.752 | 2.401 |
| Corridor Strip | 9 | extrapolation | 16 | 0.7663 | 9.096 |
| Corridor Strip | 10 | rarefaction | 8 | 0.7768 | 4.071 |
| Corridor Strip | 11 | rarefaction | 10 | 0.7767 | 5.11 |
| Corridor Strip | 12 | rarefaction | 5 | 0.7926 | 2.802 |
| **Average** | **-** | **-** | **10.7** | **0.7767** | **5.4** |
| Coffee Plantation | 1 | rarefaction | 8 | 0.7872 | 4.03 |
| Coffee Plantation | 2 | rarefaction | 8 | 0.7845 | 3.845 |
| Coffee Plantation | 4 | rarefaction | 4 | 0.7561 | 2.313 |
| Coffee Plantation | 6 | rarefaction | 19 | 0.7772 | 5.922 |
| Coffee Plantation | 7 | rarefaction | 17 | 0.7723 | 8.479 |
| Coffee Plantation | 9 | rarefaction | 6 | 0.786 | 2.948 |
| Coffee Plantation | 11 | rarefaction | 2 | 0.7503 | 1.418 |
| Coffee Plantation | 12 | rarefaction | 3 | 0.7804 | 1.819 |
| **Average** | **-** | **-** | **8.4** | **0.7742** | **3.85** |
| Pasture | 1 | rarefaction | 15 | 0.774 | 5.871 |
| Pasture | 2 | rarefaction | 2 | 0.75 | 1.5 |
| Pasture | 3 | rarefaction | 13 | 0.7694 | 6.85 |
| Pasture | 4 | extrapolation | 43 | 0.7723 | 23.173 |
| Pasture | 5 | extrapolation | 17 | 0.7742 | 8.125 |
| Pasture | 6 | rarefaction | 15 | 0.7777 | 6.655 |
| Pasture | 7 | extrapolation | 10 | 0.7867 | 6.267 |
| Pasture | 8 | extrapolation | 45 | 0.7776 | 19.604 |
| Pasture | 9 | rarefaction | 5 | 0.7862 | 2.75 |
| Pasture | 10 | rarefaction | 7 | 0.875 | 1.875 |
| Pasture | 11 | rarefaction | 12 | 0.7692 | 4.769 |
| Pasture | 12 | rarefaction | 9 | 0.8974 | 1.923 |
| **Average** | **-** | **-** | **16.1** | **0.7925** | **7.45** |
