# Supplementary material for: Variegated tropical landscapes conserve diverse dung beetle communities
Source: PeerJ. 2017 Apr 4;5:e3125. doi: 10.7717/peerj.3125 (PMC5382926; doi:10.7717/peerj.3125)
Supplement: Table S3 — Paired test values of contrast analysis to assess differences in species richness, abundance, and biomass of dung beetle among the land use and cover classes (forest fragments, forest corridors, coffee plantation and pasture), Lavras —Brazil. ∗ highlights significant results at p < 0.05. [file peerj-05-3125-s003.doc]

Table S3 Paired test values of contrast analysis to assess differences in species richness, abundance, and biomass of dung beetle among the land use and cover classes (forest fragments, forest corridors, coffee plantation and pasture), Lavras – Brazil. * highlights significant results at p < 0.05.

|  | **RICHNESS** | | | **ABUNDANCE** | | **BIOMASS** | | |
| --- | --- | --- | --- | --- | --- | --- | --- | --- |
| **Land Use** | **z** | **P** | **z** | | **P** | | **z** | **P** |
| Forest fragment *versus* Forest corridor | -1.53 | 0.4193 | -2.9 | | 0.0206* | | -3.07 | 0.0115* |
| Forest fragment *versus* Coffee plantation | -2.1 | 0.1535 | -3.1 | | 0.0107* | | -2.7 | 0.0350* |
| Forest fragment *versus* Pasture | 2.7 | 0.0360* | 6.005 | | <.0001* | | 3.4 | 0.0041* |
| Forest corridor *versus* Coffee plantation | -0.8 | 0.8622 | -0.52 | | 0.9545 | | -0.2 | 0.9980 |
| Forest corridor *versus* Pasture | 1.2 | 0.6327 | 3.15 | | 0.0089* | | 0.54 | 0.9480 |
| Coffee plantation *versus* Pasture | 0.3 | 0.9924 | 2.30 | | 0.0968 | | 0.31 | 0.9892 |
