# Supplementary material for: Variegated tropical landscapes conserve diverse dung beetle communities
Source: PeerJ. 2017 Apr 4;5:e3125. doi: 10.7717/peerj.3125 (PMC5382926; doi:10.7717/peerj.3125)
Supplement: Table S4 — Paired test values of PERMANOVA and PERMDISP analysis to assess, respectively, differences in species composition and data cloud multivariate dispersion among the land use and cover classes (forest fragments, forest corridors, coffee plantation and pasture), Lavras —Brazil. The mean values of multivariate dispersion were fragment = 39.34 (±3.24), corridor = 44.79 (±4.46), coffee = 35.46 (±3.15), and pasture = 50.88 (±2.17)—df1 = 3 and df2 = 40. [file peerj-05-3125-s004.doc]

Table S4 Paired test values of PERMANOVA and PERMDISP analysis to assess, respectively, differences in species composition and data cloud multivariate dispersion among the land use and cover classes (forest fragments, forest corridors, coffee plantation and pasture), Lavras – Brazil. The mean values of multivariate dispersion were fragment = 39.34 (±3.24), corridor = 44.79 (±4.46), coffee = 35.46 (±3.15), and pasture = 50.88 (±2.17) - df1 = 3 and df2 = 40.

|  | **PERMANOVA** | | | | **PERMDISP** | |  |
| --- | --- | --- | --- | --- | --- | --- | --- |
| **Land Use** | | **t** | **P** | **df** | **t** | **P** | |
| Forest fragment *versus* Forest corridor | | 1.6232 | 0.005 | 22 | 0.99128 | 0.411 | |
| Forest fragment *versus* Coffee plantation | | 3.6021 | 0.001 | 18 | 0.81891 | 0.501 | |
| Forest fragment *versus* Pasture | | 3.4508 | 0.001 | 22 | 2.9631 | 0.017 | |
| Forest corridor *versus* Coffee plantation | | 2.3301 | 0.001 | 18 | 1.5406 | 0.218 | |
| Forest corridor *versus* Pasture | | 2.9447 | 0.001 | 22 | 1.2282 | 0.262 | |
| Coffee plantation *versus* Pasture | | 2.7437 | 0.001 | 18 | 4.1819 | 0.003 | |
|  | |  |  |  |  |  | |
|  | |  |  |  |  |  | |
