# Supplementary material for: Variegated tropical landscapes conserve diverse dung beetle communities
Source: PeerJ. 2017 Apr 4;5:e3125. doi: 10.7717/peerj.3125 (PMC5382926; doi:10.7717/peerj.3125)
Supplement: Data S1 [file peerj-05-3125-s005.docx]

|  | Coffee Plantation | | | | | | | |
| --- | --- | --- | --- | --- | --- | --- | --- | --- |
|  | Area1 | Area11 | Area12 | Area2 | Area4 | Area6 | Area7 | Area9 |
| Ateuchus aff. carbonarius | 0 | 0 | 0 | 0 | 0 | 0 | 0 | 0 |
| Ateuchus sp. | 0 | 0 | 0 | 0 | 0 | 0 | 0 | 0 |
| Ateuchus striatulus | 0 | 0 | 0 | 0 | 0 | 0 | 0 | 0 |
| Canthidium aff. sulcatum | 0 | 0 | 1 | 0 | 1 | 0 | 0 | 0 |
| Canthidium aterrimum | 7 | 33 | 0 | 7 | 50 | 17 | 7 | 4 |
| Canthidium barbacenicum | 0 | 2 | 0 | 0 | 0 | 0 | 0 | 0 |
| Canthidium decoratum | 0 | 0 | 0 | 0 | 0 | 0 | 0 | 0 |
| Canthidium sp.1 | 0 | 2 | 0 | 0 | 0 | 0 | 0 | 0 |
| Canthidium sp.2 | 0 | 0 | 0 | 0 | 0 | 0 | 0 | 0 |
| Canthidium sp.3 | 0 | 0 | 0 | 0 | 0 | 0 | 0 | 0 |
| Canthon (Glaphyrocanthon) sp.1 | 0 | 0 | 0 | 0 | 0 | 0 | 0 | 0 |
| Canthon aff. podagricus | 0 | 0 | 0 | 0 | 0 | 0 | 0 | 0 |
| Canthon chalybaeus | 1 | 7 | 1 | 2 | 19 | 1 | 3 | 14 |
| Canthon lituratus | 0 | 0 | 0 | 0 | 0 | 0 | 0 | 0 |
| Canthon septemmaculatus histrio | 0 | 0 | 0 | 0 | 0 | 0 | 1 | 0 |
| Canthon sp.2 | 0 | 0 | 0 | 0 | 0 | 0 | 0 | 0 |
| Canthon virens | 0 | 0 | 0 | 0 | 0 | 0 | 0 | 0 |
| Coprophanaeus cyanescens | 0 | 0 | 0 | 0 | 0 | 0 | 4 | 0 |
| Coprophanaeus horus | 1 | 0 | 0 | 0 | 0 | 1 | 2 | 1 |
| Coprophanaeus spitzi | 0 | 0 | 0 | 0 | 0 | 2 | 0 | 0 |
| Deltochilum orbignyi | 0 | 0 | 0 | 0 | 0 | 0 | 0 | 0 |
| Deltochilum rubripenne | 0 | 0 | 0 | 0 | 0 | 0 | 0 | 0 |
| Deltochilum sp. | 0 | 0 | 0 | 0 | 0 | 0 | 0 | 0 |
| Dendropaemon sp. | 0 | 0 | 0 | 0 | 0 | 0 | 0 | 0 |
| Dichotomius aff. rotundigena | 0 | 0 | 0 | 1 | 1 | 0 | 0 | 0 |
| Dichotomius affinis | 0 | 0 | 0 | 0 | 0 | 0 | 0 | 0 |
| Dichotomius bicuspis | 14 | 0 | 6 | 2 | 11 | 1 | 2 | 32 |
| Dichotomius bos | 3 | 0 | 0 | 0 | 0 | 0 | 1 | 0 |
| Dichotomius carbonarius | 16 | 0 | 16 | 2 | 3 | 0 | 3 | 4 |
| Dichotomius depressicollis | 0 | 0 | 0 | 0 | 0 | 0 | 0 | 0 |
| Dichotomius fissus | 0 | 0 | 0 | 0 | 0 | 0 | 0 | 0 |
| Dichotomius mormon | 0 | 0 | 0 | 0 | 0 | 0 | 0 | 2 |
| Dichotomius nisus | 0 | 0 | 0 | 0 | 0 | 0 | 0 | 0 |
| Dichotomius sp. | 0 | 0 | 0 | 0 | 0 | 0 | 0 | 0 |
| Eurysternus caribaeus | 0 | 0 | 0 | 0 | 0 | 0 | 0 | 0 |
| Eurysternus cyanescens | 0 | 0 | 0 | 0 | 0 | 0 | 0 | 0 |
| Eurysternus hirtellus | 0 | 0 | 0 | 0 | 0 | 0 | 0 | 0 |
| Eurysternus parallelus | 0 | 0 | 0 | 0 | 1 | 0 | 0 | 0 |
| Eutrichillum hirsutum | 0 | 0 | 0 | 0 | 0 | 0 | 0 | 0 |
| Isocopris inhatus | 0 | 0 | 0 | 0 | 0 | 0 | 0 | 0 |
| Ontherus azteca | 0 | 0 | 0 | 0 | 0 | 0 | 0 | 0 |
| Onthophagus aff. hirculus | 0 | 0 | 0 | 0 | 0 | 1 | 1 | 0 |
| Onthophagus ranunculus | 0 | 0 | 0 | 0 | 0 | 1 | 0 | 1 |
| Oxysternon palaemon | 0 | 0 | 0 | 0 | 0 | 0 | 0 | 0 |
| Phanaeus kirbyi | 0 | 0 | 0 | 0 | 0 | 0 | 0 | 0 |
| Phanaeus palaeno | 2 | 0 | 0 | 0 | 0 | 0 | 0 | 0 |
| Phanaeus splendidulus | 0 | 0 | 0 | 0 | 0 | 0 | 0 | 0 |
| Pseudocanthon aff. xanthurus | 0 | 0 | 0 | 0 | 0 | 0 | 0 | 0 |
| Sybalocanthon korasaki | 0 | 0 | 0 | 0 | 0 | 0 | 0 | 0 |
| Sylvicanthon foveiventris | 0 | 0 | 0 | 0 | 0 | 0 | 0 | 0 |
| Trichillum externepunctatum | 0 | 0 | 0 | 0 | 0 | 0 | 0 | 0 |
| Uroxys sp. | 1 | 0 | 0 | 12 | 0 | 0 | 1 | 0 |

| Forest Corridor | | | | | | | | | | | |
| --- | --- | --- | --- | --- | --- | --- | --- | --- | --- | --- | --- |
| Area1 | Area10 | Area11 | Area12 | Area2 | Area3 | Area4 | Area5 | Area6 | Area7 | Area8 | Area9 |
| 0 | 0 | 0 | 0 | 0 | 0 | 1 | 0 | 0 | 0 | 0 | 0 |
| 1 | 0 | 0 | 0 | 0 | 0 | 0 | 0 | 0 | 0 | 0 | 0 |
| 0 | 0 | 0 | 0 | 0 | 0 | 0 | 0 | 0 | 0 | 0 | 0 |
| 0 | 0 | 0 | 0 | 0 | 0 | 0 | 0 | 0 | 0 | 0 | 0 |
| 7 | 1 | 7 | 0 | 2 | 6 | 2 | 1 | 0 | 0 | 0 | 2 |
| 0 | 0 | 0 | 0 | 0 | 1 | 0 | 2 | 0 | 0 | 0 | 0 |
| 0 | 0 | 0 | 0 | 0 | 0 | 0 | 0 | 0 | 0 | 0 | 0 |
| 0 | 0 | 0 | 0 | 0 | 0 | 0 | 0 | 0 | 0 | 0 | 1 |
| 0 | 0 | 0 | 0 | 0 | 0 | 0 | 0 | 0 | 0 | 0 | 0 |
| 0 | 0 | 0 | 0 | 0 | 0 | 0 | 0 | 0 | 0 | 0 | 0 |
| 4 | 0 | 0 | 0 | 1 | 0 | 45 | 0 | 0 | 17 | 0 | 0 |
| 0 | 0 | 0 | 0 | 0 | 0 | 0 | 0 | 0 | 0 | 0 | 0 |
| 0 | 0 | 0 | 0 | 0 | 2 | 0 | 0 | 0 | 0 | 0 | 0 |
| 0 | 0 | 0 | 0 | 0 | 0 | 0 | 0 | 0 | 0 | 0 | 0 |
| 0 | 0 | 0 | 0 | 0 | 0 | 0 | 0 | 0 | 0 | 0 | 0 |
| 0 | 0 | 0 | 1 | 0 | 0 | 0 | 0 | 0 | 0 | 0 | 0 |
| 0 | 0 | 0 | 0 | 0 | 0 | 0 | 0 | 0 | 0 | 0 | 0 |
| 4 | 0 | 0 | 0 | 0 | 1 | 0 | 0 | 0 | 0 | 0 | 0 |
| 0 | 0 | 0 | 0 | 0 | 0 | 0 | 0 | 0 | 0 | 0 | 0 |
| 0 | 0 | 0 | 0 | 0 | 0 | 0 | 0 | 0 | 0 | 0 | 0 |
| 0 | 0 | 0 | 0 | 0 | 0 | 0 | 0 | 0 | 0 | 0 | 0 |
| 0 | 0 | 0 | 0 | 0 | 0 | 0 | 0 | 0 | 1 | 0 | 0 |
| 0 | 0 | 1 | 0 | 0 | 0 | 0 | 0 | 0 | 0 | 3 | 0 |
| 0 | 0 | 0 | 0 | 0 | 0 | 0 | 0 | 0 | 1 | 0 | 0 |
| 0 | 3 | 0 | 0 | 0 | 0 | 0 | 0 | 0 | 0 | 0 | 0 |
| 4 | 0 | 0 | 0 | 0 | 0 | 0 | 0 | 0 | 0 | 0 | 0 |
| 5 | 4 | 2 | 2 | 2 | 0 | 1 | 0 | 2 | 5 | 11 | 0 |
| 1 | 0 | 0 | 0 | 0 | 0 | 0 | 0 | 0 | 0 | 0 | 1 |
| 21 | 4 | 1 | 7 | 1 | 0 | 0 | 0 | 9 | 9 | 0 | 0 |
| 0 | 0 | 0 | 0 | 0 | 1 | 0 | 0 | 0 | 0 | 0 | 0 |
| 0 | 0 | 0 | 0 | 0 | 0 | 0 | 0 | 0 | 0 | 0 | 0 |
| 13 | 3 | 0 | 0 | 1 | 1 | 3 | 0 | 4 | 3 | 0 | 1 |
| 0 | 0 | 0 | 0 | 0 | 0 | 0 | 0 | 0 | 0 | 0 | 0 |
| 0 | 0 | 0 | 0 | 0 | 2 | 0 | 0 | 0 | 0 | 0 | 0 |
| 6 | 0 | 0 | 0 | 0 | 0 | 0 | 1 | 1 | 0 | 1 | 0 |
| 0 | 0 | 0 | 0 | 0 | 0 | 0 | 0 | 0 | 0 | 0 | 0 |
| 1 | 0 | 0 | 0 | 0 | 0 | 1 | 0 | 2 | 2 | 0 | 0 |
| 98 | 16 | 8 | 0 | 3 | 0 | 3 | 0 | 3 | 10 | 5 | 0 |
| 0 | 0 | 0 | 0 | 0 | 0 | 0 | 0 | 0 | 0 | 0 | 0 |
| 0 | 0 | 0 | 0 | 0 | 0 | 0 | 0 | 0 | 0 | 0 | 0 |
| 1 | 0 | 2 | 0 | 0 | 0 | 0 | 0 | 1 | 0 | 0 | 0 |
| 0 | 0 | 0 | 0 | 0 | 0 | 0 | 0 | 0 | 0 | 0 | 0 |
| 0 | 0 | 0 | 0 | 0 | 0 | 0 | 0 | 0 | 0 | 0 | 0 |
| 0 | 0 | 0 | 0 | 0 | 0 | 0 | 0 | 0 | 0 | 0 | 0 |
| 0 | 0 | 0 | 0 | 0 | 0 | 0 | 0 | 0 | 0 | 0 | 0 |
| 0 | 0 | 0 | 0 | 0 | 0 | 0 | 0 | 0 | 0 | 0 | 0 |
| 0 | 0 | 0 | 0 | 0 | 0 | 0 | 0 | 0 | 2 | 0 | 0 |
| 0 | 0 | 0 | 0 | 0 | 0 | 0 | 0 | 0 | 0 | 0 | 0 |
| 26 | 0 | 7 | 0 | 4 | 1 | 31 | 0 | 18 | 9 | 0 | 0 |
| 57 | 16 | 1 | 5 | 4 | 4 | 6 | 0 | 0 | 2 | 0 | 0 |
| 0 | 0 | 0 | 0 | 0 | 0 | 0 | 0 | 0 | 0 | 0 | 0 |
| 0 | 0 | 0 | 0 | 1 | 0 | 0 | 0 | 0 | 1 | 0 | 1 |

| Forest Fragment | | | | | | | | | | | |
| --- | --- | --- | --- | --- | --- | --- | --- | --- | --- | --- | --- |
| Area1 | Area10 | Area11 | Area12 | Area2 | Area3 | Area4 | Area5 | Area6 | Area7 | Area8 | Area9 |
| 0 | 0 | 0 | 0 | 0 | 0 | 0 | 0 | 0 | 0 | 0 | 0 |
| 6 | 0 | 0 | 0 | 0 | 0 | 0 | 0 | 0 | 0 | 0 | 0 |
| 0 | 0 | 0 | 0 | 0 | 0 | 0 | 0 | 0 | 0 | 0 | 0 |
| 0 | 0 | 0 | 0 | 0 | 0 | 0 | 0 | 0 | 0 | 0 | 0 |
| 168 | 0 | 46 | 3 | 2 | 5 | 4 | 0 | 1 | 0 | 0 | 174 |
| 0 | 0 | 0 | 0 | 0 | 0 | 0 | 0 | 0 | 0 | 0 | 0 |
| 0 | 0 | 0 | 0 | 0 | 0 | 0 | 0 | 0 | 0 | 0 | 0 |
| 0 | 0 | 0 | 0 | 0 | 0 | 0 | 0 | 0 | 0 | 0 | 0 |
| 0 | 0 | 0 | 0 | 0 | 0 | 0 | 0 | 0 | 0 | 0 | 0 |
| 0 | 0 | 0 | 0 | 0 | 0 | 0 | 0 | 0 | 1 | 0 | 1 |
| 0 | 2 | 1 | 0 | 1 | 0 | 4 | 2 | 2 | 1 | 0 | 0 |
| 0 | 0 | 0 | 0 | 0 | 0 | 0 | 0 | 0 | 0 | 0 | 0 |
| 0 | 0 | 0 | 0 | 0 | 0 | 0 | 0 | 0 | 0 | 0 | 0 |
| 0 | 0 | 0 | 0 | 0 | 0 | 0 | 0 | 0 | 0 | 0 | 0 |
| 0 | 11 | 0 | 4 | 0 | 0 | 3 | 0 | 0 | 0 | 0 | 0 |
| 0 | 0 | 0 | 0 | 0 | 0 | 0 | 1 | 0 | 0 | 0 | 0 |
| 0 | 0 | 0 | 0 | 0 | 0 | 0 | 0 | 0 | 0 | 0 | 0 |
| 0 | 1 | 0 | 0 | 0 | 0 | 0 | 0 | 0 | 0 | 0 | 0 |
| 0 | 0 | 0 | 0 | 0 | 0 | 0 | 0 | 0 | 0 | 0 | 0 |
| 0 | 0 | 0 | 0 | 0 | 0 | 0 | 0 | 0 | 0 | 0 | 0 |
| 0 | 0 | 0 | 0 | 0 | 0 | 0 | 0 | 0 | 0 | 0 | 0 |
| 5 | 0 | 35 | 0 | 0 | 0 | 0 | 0 | 1 | 28 | 12 | 0 |
| 1 | 1 | 0 | 0 | 2 | 2 | 2 | 0 | 1 | 0 | 0 | 0 |
| 0 | 0 | 0 | 0 | 0 | 0 | 0 | 0 | 0 | 1 | 0 | 0 |
| 1 | 0 | 0 | 0 | 0 | 0 | 0 | 0 | 0 | 0 | 0 | 0 |
| 14 | 0 | 2 | 0 | 3 | 1 | 2 | 1 | 0 | 2 | 0 | 1 |
| 13 | 5 | 1 | 2 | 0 | 0 | 0 | 0 | 0 | 1 | 0 | 0 |
| 0 | 0 | 0 | 0 | 0 | 0 | 0 | 1 | 0 | 1 | 0 | 0 |
| 0 | 0 | 0 | 1 | 0 | 0 | 1 | 0 | 0 | 1 | 0 | 2 |
| 1 | 0 | 0 | 0 | 0 | 0 | 1 | 0 | 0 | 2 | 0 | 0 |
| 4 | 0 | 1 | 0 | 0 | 0 | 0 | 0 | 0 | 0 | 0 | 0 |
| 47 | 12 | 8 | 1 | 26 | 5 | 38 | 5 | 23 | 17 | 3 | 49 |
| 0 | 0 | 0 | 0 | 0 | 0 | 0 | 0 | 0 | 0 | 0 | 0 |
| 0 | 0 | 0 | 0 | 0 | 0 | 0 | 0 | 0 | 0 | 0 | 0 |
| 1 | 5 | 1 | 0 | 0 | 0 | 0 | 0 | 0 | 0 | 2 | 1 |
| 0 | 0 | 1 | 0 | 0 | 0 | 0 | 0 | 0 | 0 | 0 | 0 |
| 1 | 3 | 3 | 0 | 4 | 0 | 8 | 0 | 15 | 3 | 0 | 1 |
| 7 | 9 | 5 | 0 | 6 | 0 | 1 | 0 | 1 | 15 | 0 | 0 |
| 0 | 0 | 0 | 0 | 0 | 0 | 0 | 0 | 0 | 0 | 0 | 0 |
| 0 | 0 | 0 | 0 | 0 | 0 | 0 | 0 | 0 | 0 | 0 | 0 |
| 3 | 0 | 0 | 0 | 2 | 0 | 0 | 0 | 0 | 0 | 0 | 0 |
| 0 | 0 | 0 | 0 | 0 | 0 | 0 | 0 | 0 | 0 | 0 | 0 |
| 0 | 0 | 0 | 0 | 0 | 0 | 0 | 0 | 0 | 0 | 0 | 0 |
| 0 | 0 | 0 | 0 | 0 | 0 | 0 | 0 | 0 | 0 | 0 | 0 |
| 0 | 0 | 0 | 0 | 0 | 0 | 0 | 0 | 0 | 0 | 0 | 0 |
| 0 | 0 | 0 | 0 | 0 | 0 | 0 | 0 | 0 | 0 | 0 | 0 |
| 1 | 0 | 1 | 0 | 2 | 0 | 0 | 0 | 0 | 2 | 0 | 0 |
| 0 | 0 | 0 | 0 | 0 | 0 | 0 | 0 | 0 | 0 | 0 | 0 |
| 45 | 0 | 13 | 0 | 109 | 21 | 14 | 1 | 13 | 3 | 0 | 0 |
| 160 | 90 | 35 | 28 | 5 | 34 | 27 | 1 | 8 | 2 | 0 | 0 |
| 1 | 0 | 0 | 0 | 0 | 0 | 0 | 0 | 0 | 0 | 0 | 0 |
| 0 | 0 | 0 | 1 | 0 | 0 | 0 | 0 | 0 | 0 | 0 | 0 |

| Pasture | | | | | | | | | | | |
| --- | --- | --- | --- | --- | --- | --- | --- | --- | --- | --- | --- |
| Area1 | Area10 | Area11 | Area12 | Area2 | Area3 | Area4 | Area5 | Area6 | Area7 | Area8 | Area9 |
| 0 | 0 | 0 | 0 | 0 | 0 | 0 | 0 | 0 | 0 | 0 | 0 |
| 0 | 0 | 0 | 0 | 0 | 0 | 0 | 0 | 0 | 0 | 0 | 0 |
| 0 | 1 | 0 | 0 | 0 | 1 | 0 | 0 | 0 | 0 | 0 | 0 |
| 0 | 0 | 0 | 0 | 0 | 0 | 0 | 0 | 0 | 0 | 0 | 0 |
| 0 | 0 | 1 | 1 | 0 | 0 | 2 | 3 | 12 | 0 | 0 | 0 |
| 0 | 0 | 0 | 0 | 0 | 2 | 1 | 1 | 0 | 0 | 0 | 0 |
| 0 | 0 | 0 | 0 | 3 | 0 | 1 | 0 | 0 | 0 | 0 | 1 |
| 0 | 0 | 0 | 0 | 0 | 0 | 0 | 0 | 0 | 0 | 0 | 0 |
| 0 | 0 | 0 | 0 | 0 | 0 | 0 | 0 | 0 | 1 | 0 | 0 |
| 0 | 0 | 0 | 0 | 0 | 0 | 0 | 0 | 0 | 0 | 0 | 0 |
| 0 | 0 | 0 | 0 | 0 | 0 | 0 | 0 | 0 | 0 | 0 | 0 |
| 0 | 0 | 1 | 0 | 0 | 0 | 0 | 0 | 0 | 0 | 1 | 8 |
| 0 | 0 | 0 | 0 | 0 | 0 | 0 | 0 | 0 | 0 | 0 | 0 |
| 0 | 0 | 0 | 0 | 0 | 0 | 1 | 0 | 1 | 0 | 0 | 0 |
| 0 | 0 | 0 | 0 | 0 | 0 | 0 | 0 | 0 | 0 | 0 | 0 |
| 0 | 0 | 0 | 0 | 0 | 0 | 0 | 0 | 0 | 0 | 0 | 0 |
| 0 | 0 | 0 | 0 | 0 | 4 | 1 | 0 | 0 | 0 | 0 | 0 |
| 1 | 0 | 2 | 1 | 0 | 0 | 0 | 0 | 0 | 0 | 0 | 0 |
| 0 | 0 | 0 | 0 | 0 | 1 | 0 | 0 | 2 | 0 | 1 | 7 |
| 0 | 0 | 0 | 1 | 0 | 0 | 1 | 0 | 0 | 2 | 0 | 0 |
| 1 | 0 | 0 | 0 | 0 | 0 | 0 | 0 | 0 | 0 | 0 | 0 |
| 0 | 0 | 0 | 0 | 0 | 0 | 0 | 0 | 0 | 0 | 0 | 0 |
| 0 | 0 | 0 | 0 | 0 | 0 | 0 | 0 | 0 | 0 | 0 | 0 |
| 0 | 0 | 0 | 0 | 0 | 0 | 0 | 0 | 0 | 0 | 0 | 0 |
| 0 | 0 | 0 | 0 | 0 | 0 | 0 | 0 | 0 | 0 | 0 | 0 |
| 2 | 0 | 0 | 0 | 0 | 0 | 0 | 0 | 0 | 0 | 0 | 0 |
| 0 | 0 | 0 | 0 | 0 | 0 | 0 | 1 | 0 | 0 | 0 | 0 |
| 19 | 0 | 8 | 35 | 0 | 6 | 0 | 0 | 6 | 2 | 1 | 1 |
| 7 | 0 | 0 | 0 | 0 | 0 | 0 | 0 | 0 | 0 | 0 | 0 |
| 0 | 0 | 0 | 0 | 0 | 0 | 0 | 0 | 0 | 0 | 0 | 0 |
| 0 | 0 | 0 | 0 | 0 | 0 | 0 | 0 | 0 | 0 | 0 | 0 |
| 1 | 0 | 0 | 0 | 0 | 0 | 0 | 0 | 0 | 0 | 0 | 0 |
| 1 | 0 | 0 | 0 | 0 | 0 | 0 | 0 | 0 | 0 | 1 | 0 |
| 0 | 0 | 0 | 0 | 0 | 0 | 0 | 0 | 0 | 0 | 0 | 0 |
| 0 | 0 | 0 | 0 | 0 | 0 | 0 | 0 | 0 | 0 | 0 | 0 |
| 0 | 0 | 0 | 0 | 0 | 0 | 0 | 0 | 0 | 0 | 0 | 0 |
| 0 | 0 | 0 | 0 | 0 | 0 | 0 | 0 | 0 | 0 | 0 | 0 |
| 1 | 0 | 0 | 0 | 0 | 0 | 0 | 0 | 0 | 0 | 0 | 0 |
| 0 | 0 | 0 | 0 | 0 | 0 | 0 | 1 | 0 | 1 | 0 | 1 |
| 1 | 0 | 0 | 0 | 0 | 0 | 0 | 0 | 0 | 0 | 1 | 0 |
| 0 | 0 | 0 | 0 | 0 | 0 | 0 | 0 | 0 | 0 | 0 | 0 |
| 0 | 0 | 0 | 0 | 0 | 0 | 0 | 0 | 0 | 0 | 0 | 0 |
| 3 | 7 | 1 | 0 | 1 | 2 | 1 | 3 | 2 | 1 | 4 | 0 |
| 0 | 0 | 0 | 0 | 0 | 2 | 0 | 0 | 1 | 0 | 3 | 0 |
| 0 | 0 | 0 | 0 | 0 | 0 | 0 | 1 | 0 | 0 | 0 | 0 |
| 0 | 0 | 0 | 0 | 0 | 0 | 1 | 0 | 1 | 0 | 0 | 0 |
| 0 | 0 | 0 | 0 | 0 | 0 | 0 | 0 | 0 | 0 | 0 | 0 |
| 0 | 0 | 0 | 0 | 0 | 2 | 0 | 0 | 3 | 0 | 0 | 0 |
| 0 | 0 | 0 | 0 | 0 | 0 | 0 | 0 | 0 | 0 | 0 | 0 |
| 0 | 0 | 0 | 0 | 0 | 0 | 0 | 0 | 0 | 0 | 0 | 0 |
| 0 | 0 | 0 | 1 | 0 | 0 | 0 | 0 | 2 | 2 | 1 | 0 |
| 0 | 0 | 0 | 0 | 0 | 0 | 0 | 0 | 0 | 0 | 1 | 0 |
